# A New Approach for the Control and Reduction of Warpage and Residual Stresses in Bonded Wafer

**DOI:** 10.3390/mi12040361

**Published:** 2021-03-26

**Authors:** Seyed Amir Fouad Farshchi Yazdi, Matteo Garavaglia, Aldo Ghisi, Alberto Corigliano

**Affiliations:** 1 Department of Civil and Environmental Engineering, Politecnico di Milano, Piazza Leonardo da Vinci 32, 20133 Milano, Italy; seyedamir.farshchi@polimi.it (S.A.F.F.Y.); aldo.ghisi@polimi.it (A.G.); 2 STMicroelectronics, Via Camillo Olivetti 2, 20864 Agrate Brianza, Italy; matteo.garavaglia@st.com

**Keywords:** glass frit bonding, warpage, residual stress, finite element analysis

## Abstract

A geometrical modification on silicon wafers before the bonding process, aimed to decrease (1) the residual stress caused by glass frit bonding, is proposed. Finite element modeling showed that (2) by introducing this modification, the wafer out-of-plane deflection was decreased by 34%. Moreover, (3) fabricated wafers with the proposed geometrical feature demonstrated an improvement for the (4) warpage with respect to the plain wafers. A benefit for curvature variation and overall shape of the (5) bonded wafers was also observed.

## 1. Introduction

Packaging in the production of integrated circuits (ICs) and micro-electro-mechanical systems (MEMS) is carried out by exploiting different materials in order to protect the device from the external hazards and, as a consequence, to increase the reliability and the life time of the system. The process can be divided into three levels of effectiveness. At zero-level wafer-level packaging (WLP), a hermetic seal is provided for the micromachined device. At first level WLP, the amount of moisture is controlled in the device and, finally, at second level WLP, the system is also protected from electromagnetic interference and stray current [1]. Utilizing materials with different mechanical properties causes residual stress, which directly affects the system reliability.

Different techniques have been exploited for the wafer-level packaging, such as direct bonding [2], anodic bonding [3] and intermediate bonding. In the latter category, third-party materials in between of the wafers play the role of the binding component. The binder is deposited either on one or both wafers before the bonding process. Depending on the device application, different types of materials can be used, such as metals (Cu-Cu or Au-Au in thermo-compression bonding [4,5], eutectic bonding [6]), glasses (glass frit bonding [7,8]), and polymers [9,10]. It is important to recall that the packaging cost of an individual device can reach up to the 70% of the total cost [11] and therefore wafer-level packaging can significantly save money.

One of the main issues in WLP is the wafer warpage induced by residual stresses. The residual stress in the die influences the manufacturing quality in the following process steps, mostly because the system experiences different thermal load; finally, the performance of the device is also affected [12,13]. Moreover, by advancing the complexity of MEMS structures and exploiting 3D integration, the residual stress due to bonding affects the warpage of the overall stack [14]. There are several methods to measure the wafer curvature: the main ones exploit optical methods, but other approaches has been followed, see Chapter 14 in [15] or e.g., [16,17]. Numerical simulation is also pursued, to understand the influence of the materials and of the layer geometrical dimensions [18].

Several studies have been carried out in order to find a design solution able to decrease the residual stress, and, consequently, the warpage after WLP. In [19], by utilizing the analysis of variance, the most effective parameters on the warpage are found to be the substrate and the epoxy moulding compound thicknesses, while the least effective parameter is the substrate material. Liu et al. [20] proposed a bonding process with different temperature peaks at the cap and the sensor wafer. This thermal gradient approach results in a significant warpage reduction for silicon to glass wafer bonding. The layer thickness has been recognized as important also in [14], and particularly when more than two wafers are stacked; while the warping increases with the stack, the increment of wafer curvature reduces as the number of stack increases. In order to reduce the residual stress effect, appropriate material choice has been suggested, e.g., for temporary bonding materials within the carrier wafer [21]. In particular, the effective Young’s modulus of the bonding material plays an important role, the lower the modulus, the lesser the warpage. This effect combines with the mismatch in the coefficient of thermal expansion (CTE) of the materials constituting the different layers. In fact, during the bonding process, often the temperature is changing by several hundreds of degrees and, therefore, the evolution of (both thermal and mechanical) material properties during the process has to be followed to correctly estimate the overall behavior, see, e.g., [22]. If the bonding material is a fibre reinforced polymer, there is a mechanism of warpage orientation rotation dependent on a transition temperature, in turn related to the stress relaxation of the fibers in the polymer [14]. Polymeric bonding films, however, can have non trivial influence on warpage evolution [23].

Warping can also be observed after the application of the moulded package, see e.g., [24], and in processes where each single die is bonded to a substrate wafer, see e.g., [22]; in this work, we will concentrate on glass frit, a common bonding material, extensively used in the MEMS industry [7,8]. Because of the relevance of the mechanical and thermal coupled behaviour on the warping, in Section 2, the outcome of an experimental campaign on glass frit is summarized. The proposal of the modification in the wafer geometry is presented in Section 3, where numerical simulations and experimental measurements of the wafer warping are compared, before conclusions are drawn in Section 4.

## 2. Glass Frit Material for Bonding

**Figure 1:**
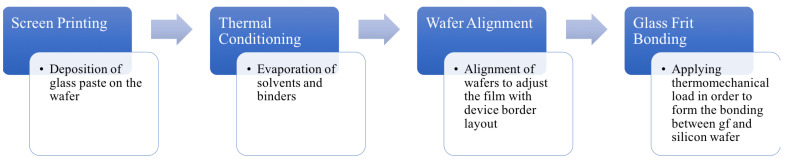
Schematic workflow of glass frit bonding.

In wafer-to-wafer glass frit bonding, a silicate or lead-silicate glass is deposited on the cap wafer via screen printing. Through a thermal conditioning process, the solvent and the binders are burnt out and a glazing process occurs at 425 ${}^{{}^{\circ}}$C. The cap wafer with the glass frit paste and the sensor wafer go through an alignment process and, afterwards, are put into the bonding chamber. The initial chamber temperature is 250 ${}^{{}^{\circ}}$C with a nitrogen ambient pressure at the scale of some hundreds millibars of nitrogen gas. When the temperature reaches 300 ${}^{{}^{\circ}}$C, the maximum mechanical load (about 10 kN) is applied on the wafers and remains constant until the end of the process. In the next step, the temperature is increased and held at 440 ${}^{{}^{\circ}}$C for 10–20 min to form the silicon–glass frit interface. The last step consists of cooling down the chamber to the initial temperature value; finally, the mechanical load is removed and the wafers are taken off the chamber to reach the ambient temperature. The schematic view of the process is presented in Figure 1.

The oxides present in the glass frit material used in this study are reported in Table 1.

A microstructural and mechanical characterization of this material under shear load has been carried out in [25], and it is briefly summarized here.

An effective, isotropic Young’s modulus *E* = 87 ± 9 GPa and an effective Poisson’s ratio ν = 0.19 ± 0.02 are obtained from nanoindentation tests. For the numerical simulation of the bonding process, since measurements of the modulus evolution with temperature are lacking, these properties are considered constant. Since numerical modeling requires also the properties of the silicon–glass frit–silicon interface, for the interface shear strength, the experimental value 1.5 × 0.3 MPa is considered (but it was never reached in the calculations), while, for the interface strength in the normal direction, a value equal to 2 MPa is assumed. The interface model used in the simulation described in Section 3 also involves shear and normal stiffnesses acting during an elastic response: again, for the shear interface stiffness, the experimental value equal to $5\times 10^{4}$ N/m${}^{2}$ is used, while, for the normal interface stiffness, a numerical calibration led to the $1\times 10^{5}$ N/m${}^{2}$ value. Moreover, glass frit bonding consists of a thermomechanical cycle aimed to provide a chemical bond between silicon and glass frit, which, in turn, leads to wafer bonding through an intermediate medium. As intrinsic sources for residual stresses can, to a given level, be annealed, thermal mismatch stresses are instead typically induced during the bonding process [26]. The temperature variation in the process causes in fact an excessive stress at the interface of each layer, especially at the cooling stage of the bonding process due to thermal expansion coefficient (CTE) mismatch: the CTE of glass frit with respect to the silicon is in fact significantly higher, namely at room temperature it is $\alpha_{glassfrit}=7.00\times 10^{-6}$ ${}^{{}^{\circ}}$C${}^{-1}$ and $\alpha_{silicon}=2.46\times 10^{-6}$ ${}^{{}^{\circ}}$C${}^{-1}$. As a first approximation, the thermal strain at the silicon–glass frit interface can be obtained by the following equation:

(1) $$\varepsilon_{th}=\left(\alpha_{glassfrit}-\alpha_{silicon}\right)\times \Delta T$$

where $\varepsilon_{th}$ is the thermal strain, $\alpha_{\left(\cdot \right)}$ is the thermal expansion coefficient for the correspondent material, and Δ*T* is the temperature difference between the different stages of the bonding. It is worthwhile to emphasize that this linear relationship holds true when the temperature gradient is low, as in this case, since the temperature variation rate is between 10 to 20 ${}^{{}^{\circ}}$C/min. However, during the transition, the Young’s modulus is varying as well, and its variation curve is still unclear. Therefore, Equation (1) should be considered only as a qualitative guess in this context, evidencing the main variables playing a role in the process, and not (unfortunately) as a quantitative estimation.

**Figure 2:**
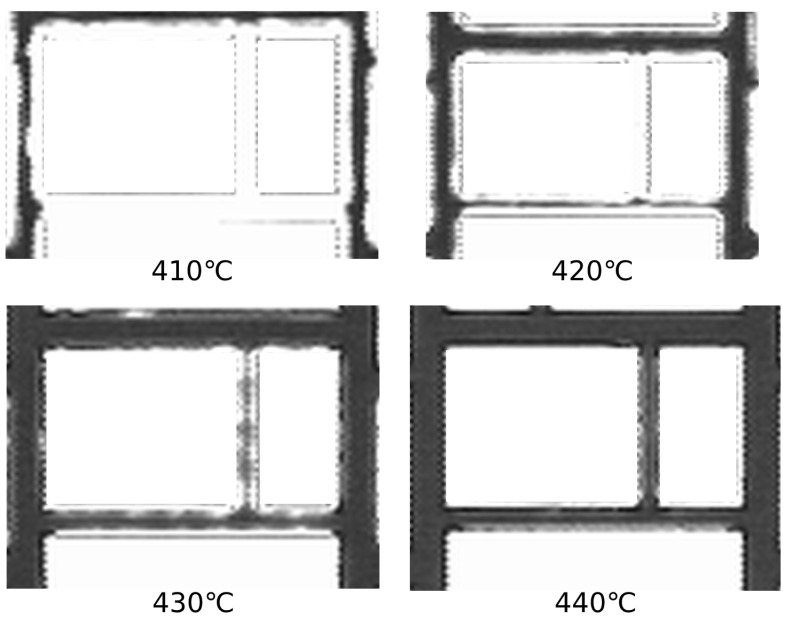
Scanning acoustic microscopy images of glass frit lines at different temperatures during the bonding. Thicker lines correspond to better bonding surfaces.

A trivial solution to decrease the effect of CTE mismatch would be to decrease the bonding temperature, but this idea cannot be pursued here because in glass frit bonding, due to the film high surface roughness and in order to compensate the lack of contact points, partial melting and viscous flow of the glass frit play an important role and they have a direct effect on the bonding yield. In Figure 2, scanning acoustic microscopy (SAM) images of glass frit bonding with different maximum bonding temperature are shown (each image has the same resolution). Regions filled in black indicate wafer bonded parts, while, in the white regions, since there is no acoustic wave reflection, there is a discontinuity between silicon and glass frit (i.e., no bonding). It can be concluded that the maximum bonding quality is obtained if the bonding temperature is at or above 440 ${}^{{}^{\circ}}$C. Moreover, decreasing the cooling rate to maintain the strain variation in a quasi-equilibrium state is a solution not applicable, since it elongates the bonding time and it is not economically efficient.

**Figure 3:**
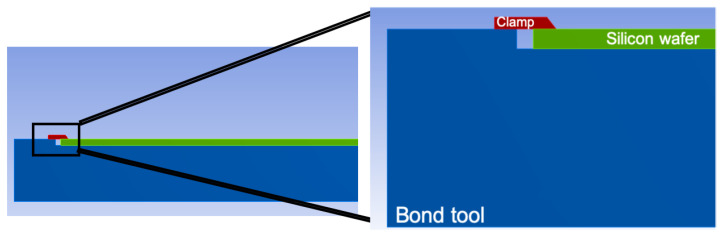
Lateral view and zoom of the wafer system gripped with tool clamps.

Mechanical constraints applied on the silicon wafers during the bonding by means of bond tool clamps also affect the residual stress. These clamps are shown in Figure 3: they help with maintaining the silicon wafers fixed in the alignment process as well as transporting them into the bonding chamber. These clamps are placed along the radial direction of the wafers. As it can be guessed and as a finite element model of glass frit bonding shows, see [25], wafers experience a high stress level during the bonding process at the regions fixed by these clamps.

## 3. Modification of Wafer Geometry

Reducing the effect of CTE mismatch by changing the involved materials is out of scope; therefore, to diminish the wafer warpage, the effect of mechanical grips shown in Figure 3 must be decreased. As it is done typically in other compressed circular plates, a workaround to mitigate the severity of residual stress consists of adding a hole at the center of the wafer because this boundary condition would offer a free path to the silicon thermal expansion.

**Figure 4:**
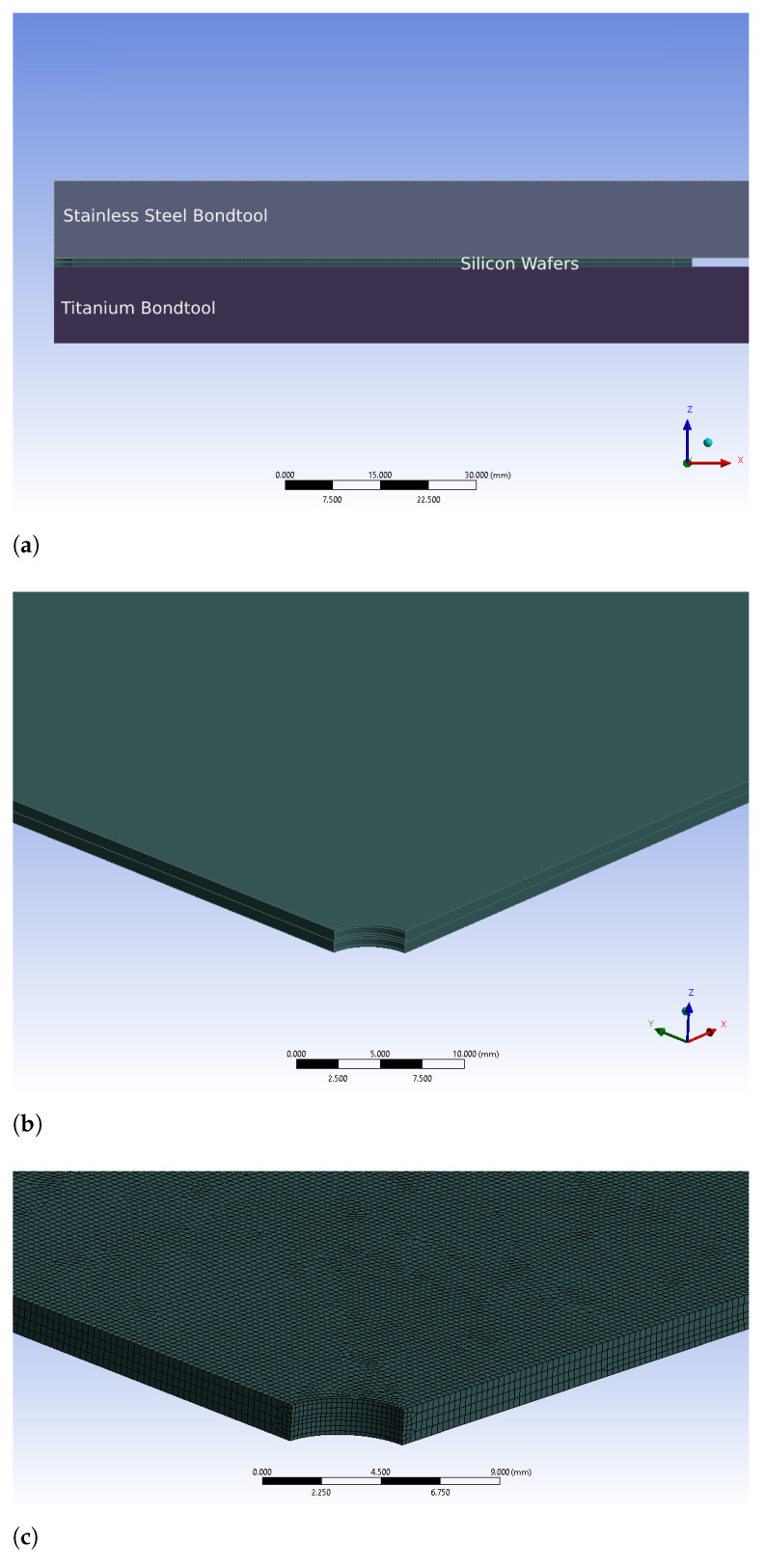
FE model of the geometrical modified silicon wafers under the glass frit bonding process. (**a**) Bonding chamber model configuration; (**b**) Wafers with central hole; (**c**) FE of wafers with central hole geometry.

In order to study the effect of the above-mentioned hypothesis as well as to obtain the optimal geometrical parameters, the new geometry is introduced into the finite element model of glass frit bonding. Two (001), 725 μm-thick monocrystalline silicon wafers with anisotropic elasticity are considered. They are put into contact, while the bottom surface of the lower wafer is in turn at unilateral contact with a titanium bond tool. The upper wafer is also limited by another tool, made of stainless steel (Figure 4a). Titanium and stainless steel temperature-varying properties are taken from [27,28]; the silicon properties are instead obtained from [29,30]. The glass frit intermediate layer is modeled by means of zero-thickness interface finite elements. Additional details can be found in [25]. A pressure is applied to this tool and the mechanical load is transferred to the top surface of the upper wafer. Mechanical clamps, as mentioned in Section 2, restrain the wafer along its diameter. A temperature history similar to [31] is applied to the bond tools and is transferred to the wafers.

### 3.1. Numerical Results

**Figure 5:**
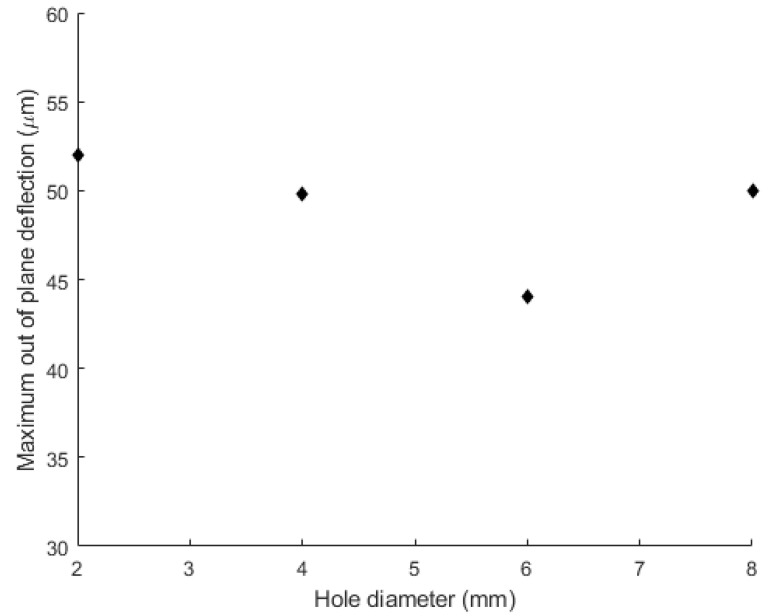
Out-of-plane deflection vs. the diameter of central hole.

**Figure 6:**
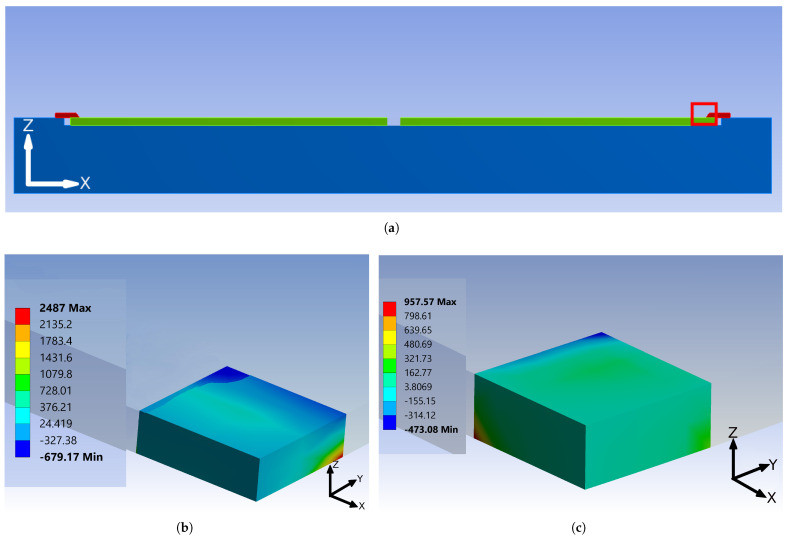
Map of maximum principal stresses, in MPa, at the wafer clamped region. (**a**) Position of clamps with respect to the central hole; (**b**) Wafers without central hole; (**c**) Wafers with central hole (6 mm diameter).

To investigate the effect of the central hole, a cylindrical volume with the diameter ranging from 2 to 8 mm is removed from the wafers (Figure 4b); then, the bonding sequence is simulated for the system, in terms of thermomechanical loads and constraints. As shown in Figure 5, the optimal diameter, i.e., inducing the minimum out-of-plane deflection, corresponds to 6 mm. In addition, this modification shows a beneficial effect in terms of stress reduction for the wafers regions under the bond tool clamp (Figure 6).

**Figure 7:**
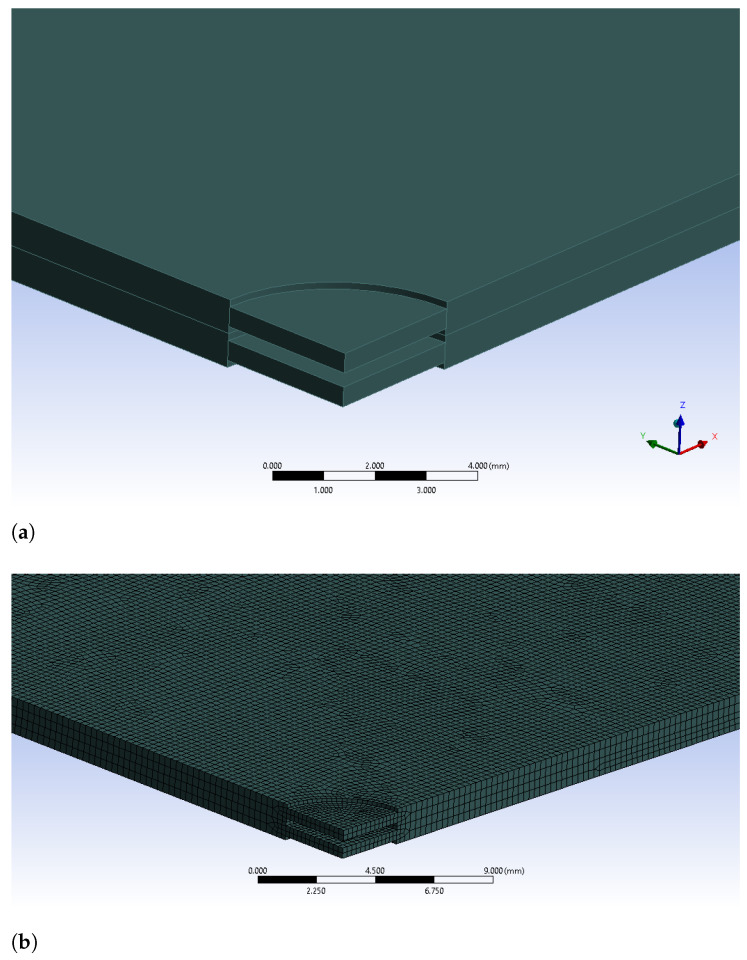
Geometrical model of two wafers with the reduced-thickness configuration. (**a**) Wafers with reduced thickness zone at center; (**b**) FE mesh for reduced thickness silicon wafers.

However, a simple central hole appears not feasible because it would cause depressurization (i.e., the suction of the wafer towards the bond tool, necessary during the alignment process and obtained by removing air underneath the wafer, would be made impossible) and it would prevent the wafer from sticking to the bond tools; without this restraint, the wafer could instead slip during the motion from the alignment stage to the bonding chamber. Conversely, if a simple thickness reduction at the center is implemented, then a similar result could be expected. Henceforth, a reduced-thickness region at the wafer center is defined with a circular shape, and it is applied on both sides of all of the silicon wafers which are supposed to be bonded. The isometric view of the wafers with this geometrical feature as well as the finite element mesh for this particular geometry are presented in Figure 7.

**Figure 8:**
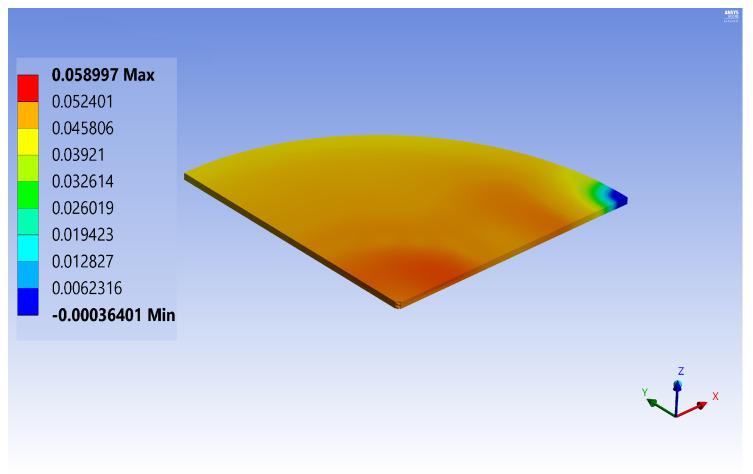
Numerical simulation: out-of-plane deflection (in mm) after glass frit bonding for wafers.

The geometry reduction diameter is chosen according to the results of the aforementioned parametric analysis (6 mm) and the thickness is 425 μm. In this case, the maximum deflection is reduced from 89 to 58 μm (see Figure 8), decreasing the wafer warpage after the bonding process of about 34%.

### 3.2. Experimental Results

**Figure 9:**
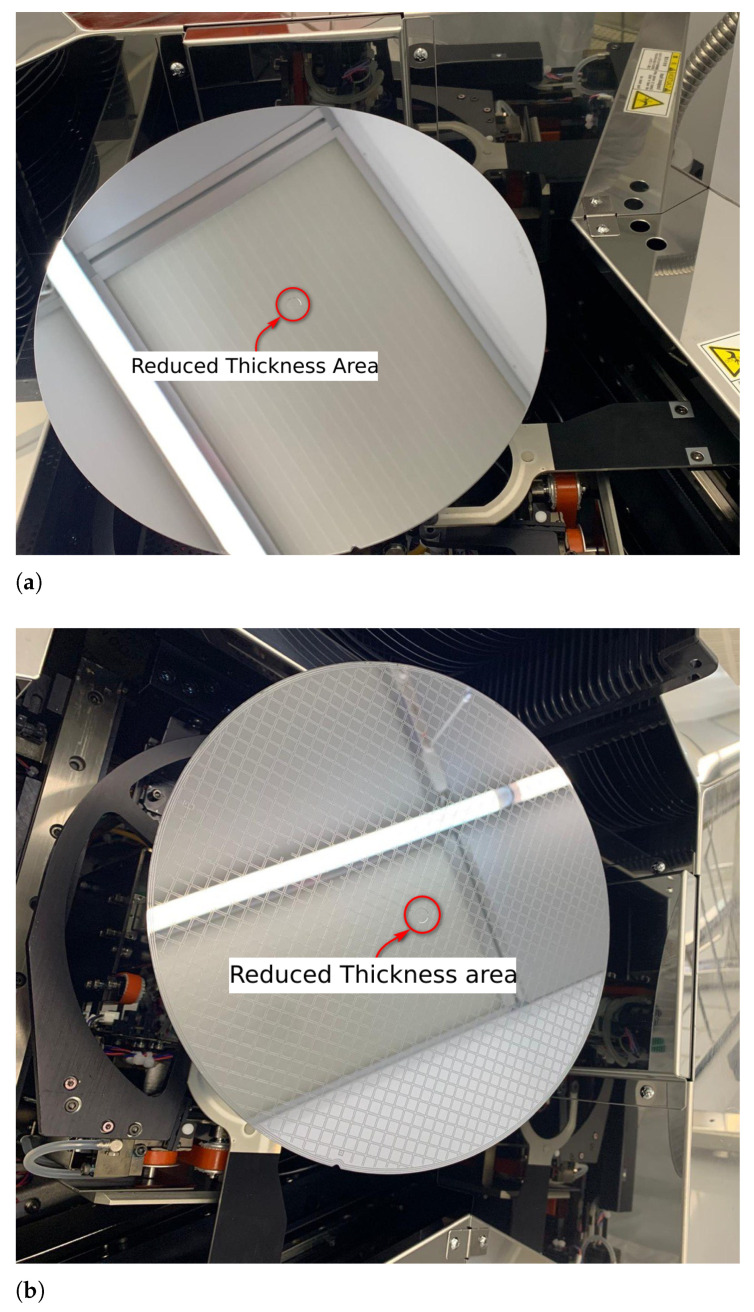
Silicon wafers fabricated with the reduced thickness geometry. (**a**) The reduced thickness area on the supposed to be the microsystem wafer; (**b**) The reduced thickness area on the cap wafer (with glass frit lines).

To verify the numerical results and the beneficial effect of the reduced thickness region on residual stress in glass frit bonding, 8-inch monocrystalline (001) silicon wafers with the reduced thickness region with the dimensions described in the previous section were fabricated (see Figure 9) by a micromachining process and bonded through glass frit (hereafter called *reduced* specimen). In addition, a set of plain silicon wafers were bonded by the same process in order to set a reference to study the effect of micromachining process on the residual stress (hereafter called *reference* specimen).

**Figure 10:**
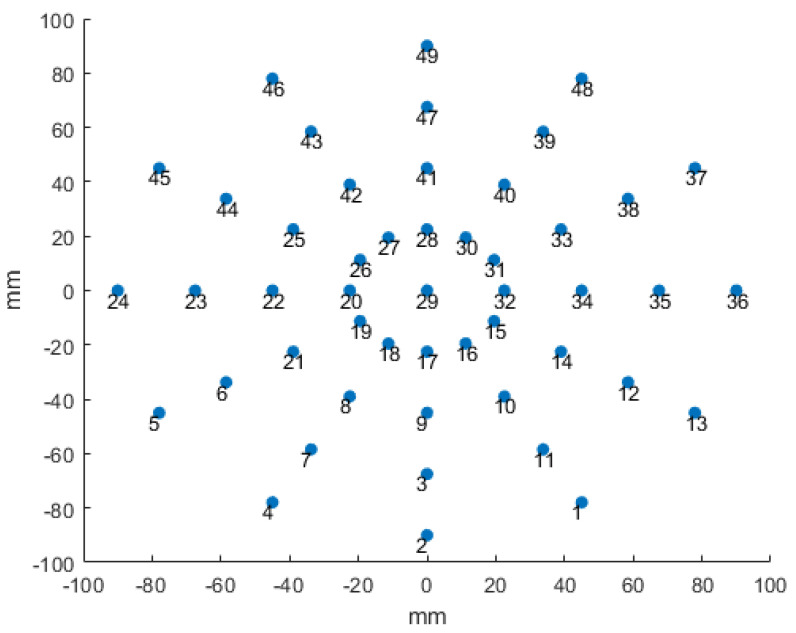
Coordinates of the points where deflection is measured.

After the bonding process, the out-of-plane deflection is measured both for reduced and reference specimens at 49 different points on the bonded wafer surface. These experimental values were obtained by using a laser beam that retrieved the height of the points on the wafer with respect to a reference plane. The coordinates of these points are presented in Figure 10.

**Figure 11:**
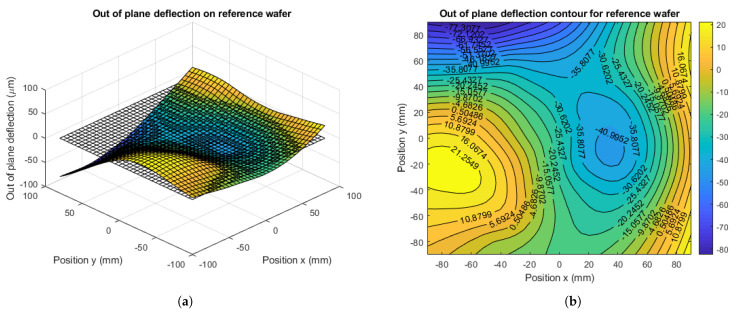

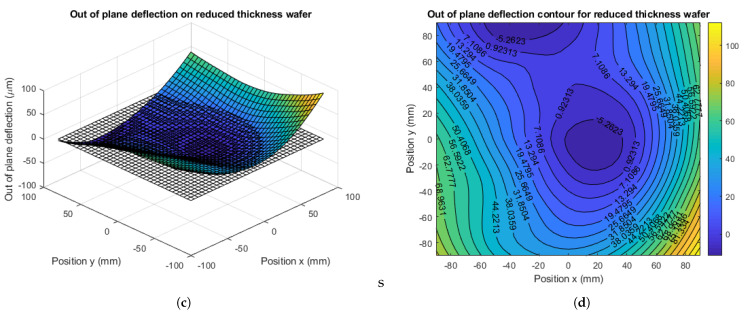
Out-of-plane experimental measurements. (**a**) Wafer surface for the reference specimen; (**b**) Isoline contour of out-of-plane deflection for the reference specimen; (**c**) Wafer surface for the reduced specimen; (**d**) Isoline contour of out-of-plane deflection for the reduced specimen.

By interpolating the out-of-plane deflection data, a silicon wafer virtual surface after the glass frit bonding is created. Figure 11a,c depict the bonded wafer surface with respect to the zero-stress state plane and Figure 11b,d show the isoline contour plots of the out-of-plane deflection for the reference and for the reduced specimens, respectively.

**Figure 12:**
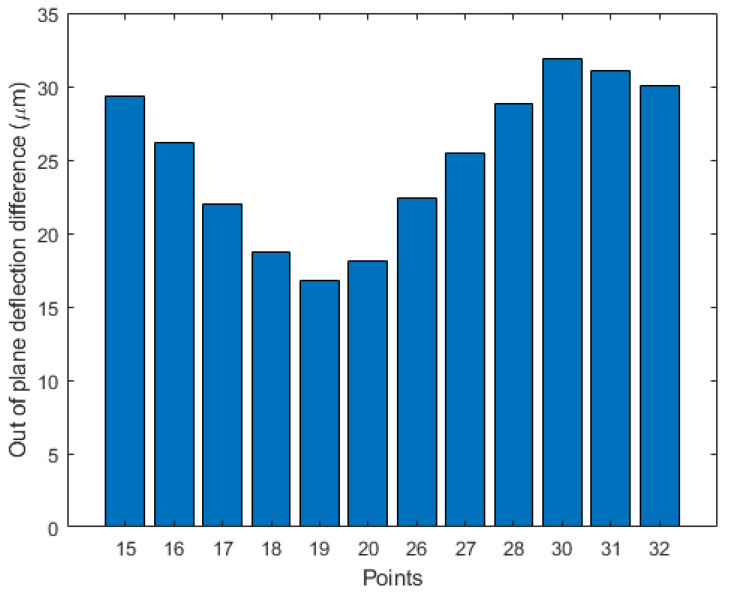
Difference in out-of-plane deflection between the reference and reduced wafers for selected points.

As obtained in the numerical model, experimental results also show a warpage decrease in the reduced specimen, especially in the central regions. By focusing on points 15–20, 26–28, 30–32, see Figure 10, which are outside of the reduced thickness region, the out-of-plane deflection has been reduced up to 32 μm, see Figure 12. Therefore, the experimental data confirm the trend shown by the numerical model and the advantage of a reduced thickness region on decreasing the residual stress imposed by mechanical constraints.

**Figure 13:**
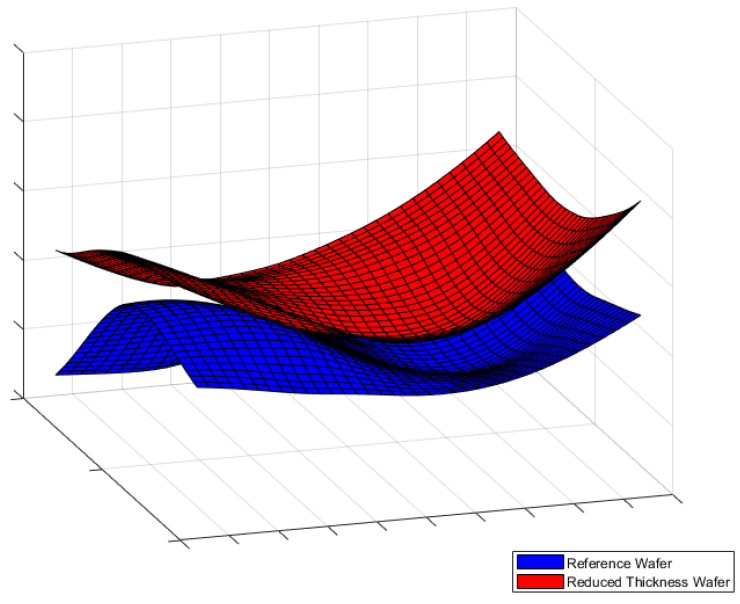
Overall shape comparison for reference and reduced specimen.

Moreover, the new geometrical feature guarantees another gain, related to the overall shape of the bonded wafers. As presented in Figure 11a,b, the surface of the reference specimen shows significant curvature variations along the surface; on the other hand, the curvature of reduced specimen remains more uniform, see Figure 11c,d. The qualitative comparison of the overall wafer shapes is shown in Figure 13.

It is worthwhile to emphasize that the depth of the trench in this work is chosen as a conservative value, in order to avoid any rupture in the wafer during the bonding process. By assuming that the diameter of the region as constant, the deeper the trench, the more space available for silicon to release excessive stress. Previous studies show that the mechanical properties of silicon is highly dependent on two factors: thickness and roughness [32]. Since the reduced thickness region is introduced by dry etching, the surface of the region would be at its best. Hence, the depth could be increased as long as the material in this region can bear the mechanical loads and deflections during the bonding process.

Finally, it is worthwhile to mention that, since the elastic stiffness of monocrystalline silicon is generally different along different directions, the optimal shape of the reduced thickness would be elliptical, considering the anisotropic mechanical properties of the wafer.

## 4. Conclusions

In this paper, first, the sources and parameters causing the residual stress in glass frit bonding, one of the renowned methods in MEMS vacuum encapsulation, are recalled. A geometrical modification to decrease the effect of mechanical constraints is then introduced. Numerical model results show about 34% warpage reduction for wafers having this feature after bonding. Modified wafers according to the proposed idea are fabricated and compared with a plain silicon wafer after glass frit bonding. Experimental data confirm the beneficial effect of the geometrical modification, as well as an improvement on the overall shape of wafers in terms of the uniformity of the wafer curvature. The outcome of this research suggests a trade-off between losing devices at the central part of the wafer and gaining dies with less residual stress and shape distortion.

## Figures and Tables

**Table 1:** Contained oxides and their percentage in the glass frit paste.

Oxide	Composition Mean Percentage (%)
SiO${}_{2}$	10 ± 3
Al${}_{2}$O${}_{3}$	6 ± 3
MgO	1.7 ± 0.5
CaO	0.1 ± 0.05
BaO	0.3 ± 0.1
PbO	65 ± 4
B${}_{2}$O${}_{3}$	13 ± 3
ZnO	2.6 ± 0.5
Na${}_{2}$O	0.2 ± 0.1

## References

[1] Tilli M., Paulasto-Krockel M., Motooka T., Lindroos V. (2015). Handbook of Silicon Based MEMS Materials and Technologies.

[2] Shimbo M., Furukawa K., Fukuda K., Tanzawa K. (1986). Silicon-to-silicon direct bonding method. J. Appl. Phys..

[3] Lee B., Seok S., Chun K. (2003). A study on wafer level vacuum packaging for MEMS devices. J. Micromech. Microeng..

[4] Yang Y.T., Chou T.C., Yu T.Y., Chang Y.W., Huang T.Y., Yang K.M., Ko C.T., Chen Y.H., Tseng T.J., Chen K.N. (2017). Low-Temperature Cu–Cu Direct Bonding Using Pillar–Concave Structure in Advanced 3D Heterogeneous Integration. IEEE Trans. Component. Packag. Manuf. Technol..

[5] Tofteberg H.R., Schjølberg-Henriksen K., Fasting E.J., Moen A.S., Taklo M.M., Poppe E.U., Simensen C.J. (2014). Wafer-level Au—Au bonding in the 350–450 C temperature range. J. Micromech. Microeng..

[6] Chidambaram V., Wickramanayaka S. (2015). Al-Ge diffusion bonding for hermetic sealing application. J. Electron. Mater..

[7] Knechtel R. (2005). Glass frit bonding: An universal technology for wafer level encapsulation and packaging. Microsyst. Technol..

[8] Knechtel R., Wiemer M., Frömel J. (2006). Wafer level encapsulation of microsystems using glass frit bonding. Microsyst. Technol..

[9] Gong Z., Zhang Y., Guo X., Liu Z. (2018). Wafer-level packaging method for RF MEMS applications using pre-patterned BCB polymer. Micromachines.

[10] Civale Y., Tezcan D.S., Philipsen H.G., Duval F.F., Jaenen P., Travaly Y., Soussan P., Swinnen B., Beyne E. (2011). 3D wafer-level packaging die stacking using spin-on-dielectric polymer liner through-silicon vias. IEEE Trans. Component. Packag. Manuf. Technol..

[11] Madou M. (2002). Fundamentals of Microfabrication: The Science of Miniaturization.

[12] Dutta S., Imran M., Pal R., Jain K.K., Chatterjee R. (2011). Effect of residual stress on RF MEMS switch. Microsyst. Technol..

[13] Li P., Gao S., Liu H., Liu J., Shi Y. (2013). Effects of package on the performance of MEMS piezoresistive accelerometers. Microsyst. Technol..

[14] Kim Y., Kang S.K., Kim S.D., Kim S.E. (2012). Wafer warpage analysis of stacked wafers for 3D integration. Microelectron. Eng..

[15] Corigliano A., Ardito R., Comi C., Frangi A., Ghisi A., Mariani S. (2017). Mechanics of Microsystems.

[16] Trujillo J., Rodrigues J.M.R. Wave Front Phase Imaging of Wafer Warpage. Proceedings of the 2018 International Wafer Level Packaging Conference (IWLPC).

[17] Schiavone G., Murray J., Smith S., Desmulliez M.P.Y., Mount A.R., Walton A.J. (2016). A wafer mapping technique for residual stress in surface micromachined films. J. Micromechan. Microeng..

[18] Abdelnaby A.H., Potirniche G.P., Barlow F., Elshabini A., Groothuis S., Parker R. Numerical simulation of silicon wafer warpage due to thin film residual stresses. Proceedings of the IEEE Workshop on Microelectronics and Electron Devices (WMED).

[19] Lee H.J., Park S.M., Park S.J. (2016). Minimization of warpage for wafer level package using response surface method. Int. J. Precis. Eng. Manuf..

[20] Liu D., Liu H., Liu J., Hu F., Fan J., Wu W., Tu L. (2020). Temperature Gradient Method for Alleviating Bonding-Induced Warpage in a High-Precision Capacitive MEMS Accelerometer. Sensors.

[21] Wu Q., Liu X., Han K., Bai D., Flaim T. Temporary Bonding and Debonding Technologies for Fan-out Wafer-Level Packaging. Proceedings of the IEEE 67th Electronic Components and Technology Conference.

[22] Chiu T.C., Yeh E.Y. (2018). Warpage simulation for the reconstituted wafer used in fan-out wafer level packaging. Microelectron. Reliab..

[23] Kim C., Lee T.I., Kim M.S., Kim T.S. (2017). Mechanism of warpage orientation rotation due to viscoelastic polymer substrates during thermal processing. Microelectron. Reliab..

[24] Huber S., Dijk M., Walter H., Wittler O., Thomas T., Lang K.D. (2014). Improving the FE simulation of molded packages using warpage measurements. Microelectron. Reliab..

[25] Farshchi Yazdi S.A.F., Garavaglia M., Ghisi A., Corigliano A. Glass frit bonding: Characterization, modeling and simulation.

[26] Freund L.B., Suresh S. (2003). Thin Film Materials. Stress, Defect Formation and Surface Evolution.

[27] Welsch G., Boyer R., Collings E. (1993). Materials Properties Handbook: Titanium Alloys.

[28] Davis J.R. (1994). Stainless Steels.

[29] Bourgeois C., Steinsland E., Blanc N., De Rooij N. Design of resonators for the determination of the temperature coefficients of elastic constants of monocrystalline silicon. Proceedings of the International Frequency Control Symposium, IEEE.

[30] Hopcroft M., Nix W., Kenny T. (2010). What is the Young’s Modulus of Silicon?. J. Microelectromech. Syst..

[31] Wu G., Xu D., Xiong B., Wang Y., Wang Y., Ma Y. (2012). Wafer-level vacuum packaging for MEMS resonators using glass frit bonding. J. Microelectromech. Syst..

[32] Barnat S., Frémont H., Gracia A., Cadalen E. (2012). Evaluation by three-point-bend and ball-on-ring tests of thinning process on silicon die strength. Microelectron. Reliab..

